# The transaction behavior of cryptocurrency and electricity consumption

**DOI:** 10.1186/s40854-023-00449-7

**Published:** 2023-01-18

**Authors:** Mingbo Zheng, Gen-Fu Feng, Xinxin Zhao, Chun-Ping Chang

**Affiliations:** 1 School of Economics and Management, Chang’an University, Xi’an, 710064 Shaanxi People’s Republic of China; 2 School of Economics and Finance, Xi’an Jiaotong University, Xi’an, 710061 Shaanxi People’s Republic of China; 3 Department of Marketing Management, Shih Chien University, Kaohsiung, 84550 Taiwan

**Keywords:** Transaction behavior, Electricity consumption, Cryptocurrency, Comovement, E31, G12

## Abstract

Rapidly increasing cryptocurrency prices have encouraged cryptocurrency miners to participate in cryptocurrency production, increasing network hashrates and electricity consumption. Growth in network hashrates has further crowded out small cryptocurrency investors owing to the heightened costs of mining hardware and electricity. These changes prompt cryptocurrency miners to become new investors, leading to cryptocurrency price increases. The potential bidirectional relationship between cryptocurrency price and electricity consumption remains unidentified. Hence, this research thus utilizes July 31 2015–July 12 2019 data from 13 cryptocurrencies to investigate the short- and long-run causal effects between cryptocurrency transaction and electricity consumption. Particularly, we consider structural breaks induced by external shocks through stationary analysis and comovement relationships. Over the examined time period, we found that the series of cryptocurrency transaction and electricity consumption gradually returns to mean convergence after undergoing daily shocks, with prices trending together with hashrates. Transaction fluctuations exert both a temporary effect and permanent influence on electricity consumption. Therefore, owing to the computational power deployed to wherever high profit is found, transactions are vital determinants of electricity consumption.

## Introduction

Since emerging in 2009, cryptocurrencies have received significant attention (Mensi et al. 2019; Sigaki et al. 2019; Cui and Maghyereh 2022), with investors and news media emphasizing their initial skyrocketing prices (Rauchs et al. 2018). Bitcoin, the most popular cryptocurrency, was priced in 2011 at $0.296 (US) per Bitcoin, closing at $11,583 on July 12, 2019 after falling back from its peak of over $20,000. Ethereum, another well-known cryptocurrency, also experienced significant gains, increasing from $0.734 to $273 on January 1, 2015 and July 12, 2019, respectively. New cryptocurrencies like Terra have once again pushed interest and expectations onto them, with investors hoping for another round of strong price growth in the cryptocurrency market.

The rapid rise in cryptocurrency transaction behaviors is accompanied by growth in the network hashrate and electricity consumption. Electricity consumption from cryptocurrency mining has maintained an upward trend and has grown over time (Stoll et al. 2019). Krause and Tolaymat (2018) show that the mining 1 Bitcoin required 1,005 kWh in the beginning of 2016 but rose to 60,461 kWh in June 2018. According to the Digiconomist,^1^ the electricity consumption of Bitcoin roughly equates to 131.26 TWh annually, which can be compared to power consumption in Argentina. Cryptocurrencies aside from Bitcoin also consume enormous amounts of electricity during validation and transactions (Li et al. 2019). Gallersdorfer et al. (2020) note that cryptocurrencies besides Bitcoin comprise about 33% of electricity consumption in the cryptocurrency market. The fast-growing energy consumption of cryptocurrency has led to concerns about its environmental effect and carbon footprint (Jiang et al. 2021; Sarkodie et al. 2022; de Vries et al. 2022). Mora et al. (2018) show that cryptocurrencies can potentially raise global temperatures by over 2 Celsius within less than three decades.

Owing to the cryptocurrency market’s massive electricity consumption, some studies examine its influencing factors through trading volume, return, and financial indicators. Corbet et al. (2021) find that Bitcoin returns positively influence the price volatility of Chinese and Russian electricity companies. Huynh et al. (2022) study how return and volume interact with Bitcoin energy consumption. Based on bidirectional causality, energy usage and Bitcoin return are correlated. Huynh et al. (2022) further find that Bitcoin volume exhibits higher connectedness with energy usage. Sarkodie et al. (2022) agrees with Huynh et al. (2022) who highlight that Bitcoin trade volume can boost long-term energy consumption. Erdogan et al. (2022) further focus on the asymmetric relationship between cryptocurrency and environmental sustainability and show that the positive shock of Bitcoin demand exerts a causal influence on environmental degradation.

With increasing electricity consumption and transaction behavior in practice, their cointegration remains undetermined. Does cryptocurrency transaction behavior co-move with electricity consumption? Related literature neglects the comovement and causal relation between transaction behaviors and electricity consumption (Hayes 2017; Fantazzini and Kolodin 2020). Huynh et al. (2022) show that transaction behavior interact with cryptocurrency energy consumption. Schinckus et al. (2022) suggest that cryptocurrency hashrates positively cointegrate with electricity consumption. Hence, in this study, we explore the long-run dynamics between cryptocurrency transaction behavior and electricity consumption. Indeed, understanding interaction dynamics between transaction behavior and electricity consumption could help investors make investment decisions and governments develop regulatory policy (Wang et al. 2022c). Our understanding of the causal relationship between price and electricity consumption must be improved by utilizing reliable and advanced statistical approaches.

Cryptocurrency transaction behavior theoretically affects electricity consumption and vice versa. On the one hand, cryptocurrency miners must provide computational power to the mining system for mining, validations, and transactions (Baldwin 2018; Stoll et al. 2019; Zięba et al. 2019). Increased computational power with proof-of-work algorithm prompts market participants to add more cryptocurrency mining investment, bringing significant growth in network hashrates and subsequent electricity consumption (Georgoula et al. 2015; Hayes 2017; Kjærland et al. 2018; Kristoufek 2015). On the other hand, greater electricity consumption requirements increase the cost of special hardware and electricity needed by cryptocurrency miners, reducing the obtained rewarded coins from mining and crowding out miners. Das and Dutta (2020) state that energy costs exert a significant effect on the exit decision of miners and serve as a weakness. As these miners lose their channel for investing in cryptocurrency through mining activities, they shift their role to become investors in the cryptocurrency market and enlarge the demand for cryptocurrencies, which exerts a promoting effect on their transactions. Hence, transaction behaviors tend to be associated with network hashrates and electricity consumption theoretically in a bidirectional way in the cryptocurrency market.

This study thus first utilizes data of 13 cryptocurrencies to investigate the comovement relationship and short- and long-run causal effects among variables. The cryptocurrency market has witnessed large fluctuations and exhibits structural breaks (Canh et al. 2019; Sahoo 2021), which may affect the relationship between transaction behaviors and electricity consumption. Specifically, we consider structural breaks induced by external shocks (e.g., passing of recognition law for Bitcoin as official payments in Japan and Bitcoin’s hard fork in July 2017) in the stationary analysis and comovement relationship during the period of concern. This study further uses the vector error correction model to identify the daily (short-run) and persistent (long-run) causal effects between transaction behaviors and electricity consumption. Our findings from July 2015 to July 2019 show that transaction behavior and electricity consumption follow a very stationary process without unit root after considering external shocks. Hence, market participants could infer the future trend of the two and conveniently develop an investment strategy. Moreover, our evidence indicates that cryptocurrency electricity consumption presents a trend of moving together with transaction behavior during the sample time. Furthermore, transaction fluctuations not solely exert daily effects but also permanent influences on electricity consumption. Conversely, the effects of electricity consumption on transactions converge to zero quickly.

Our results suggest that the government should examine transaction dynamics in the cryptocurrency market as transactions drive electricity consumption growth (Fu et al. 2022) and, hence, generate environmental costs like CO_2_ emissions from the electricity needed for cryptocurrency mining. Our stationary analysis results also suggest that cryptocurrency market investors need not adjust their investment when facing market fluctuations as external shocks are short-lived, and market forces can pull prices back toward equilibrium level.

This research offers two improvements for extant literature. First, it employs the cointegration test with structural breaks included and cross-sectional dependence to examine comovement between transaction behaviors and electricity consumption. Previous studies have discussed the nexus of Bitcoin return and electricity consumption (Huynh et al. 2022) or cointegration relationship between them (Fantazzini and Kolodin 2020). However, no one has considered a structural break and cross-sectional dependence when exploring cointegration between cryptocurrency transaction behaviors and electricity consumption. Disregarding structural break and cross-sectional dependence may lead to misleading results. Our research advances examines comovement between them using the panel cointegration technique with structural breaks and cross-sectional dependence. Second, we identify short- and long-run causalities between transaction behaviors and electricity consumption. While causality between financial determinants and energy consumption of cryptocurrency has been explored (Sarkodie et al. 2022), structural breaks are not included in the causality analysis (Fantazzini and Kolodin 2020). Our study covers short- and long-run causalities simultaneously and includes breaks obtained from the cointegration test, which can support the credibility of causality.

The reminder of this paper is structured as follows. "Methods and data" section describes the empirical setting, including the data sources, variable definitions, and the model. "Results" section presents empirical results. “Discussion and conclusion” section offers discussion and conclusion.

## Methods and data

### Panel unit toot test with structural breaks

In financial economics, the panel unit root test is widely used to investigate the stability of a series (Peng et al. 2022; Yin et al. 2022; Charfeddine and Khediri 2016; Jung and Maderitsch 2014). As booms and busts occur in the cryptocurrency market, checking the stability of transaction behaviors and electricity consumption should consider potential structural breaks (Bouri et al. 2019; Cheah and Fry 2015). We employed the panel Lagrange multiplier (LM) unit root test (Im et al. 2005) to determine the stationarity of the series of cryptocurrency transaction behaviors and electricity consumption. The panel LM unit root allows us to explore the stability of series during structural breaks induced by external shocks and can discover the changing direction of level shifts.

Our analysis examined structural breaks and their significance. The null hypothesis of the panel LM unit root test is that the series have a unit root and are not stationary (Wen et al. 2021; Wang et al. 2022a). The data-generating process of the panel LM unit root test is given by the following:

1 $$y_{it} = \gamma_{1i} + \gamma_{2i} t + \delta_{i} TB_{it} + u_{it} ,$$

where *y*_*it*_ denotes the concerned variable, including cryptocurrency transaction behaviors and electricity consumption; t = 1,…,T represents the time period; i = 1,…N represents the number of cryptocurrency; *u*_*it*_ = *ϕ*_*i*_*u*_*it-1*_ + *ε*_*it*_; *TB*_*it*_ = 1 (time break) for t > *BP*_*i*_ and 0 otherwise; *BP*_*i*_ (break point) is the estimated break for cryptocurrency i; and *ε*_*it*_ represents the error term. ${\delta }_{i}$ denotes the coefficient of break. *ϕ*_*i*_ represents the correlation parameter for the error term. We constructed a panel LM unit root test statistics by averaging univariate LM test statistics for each cryptocurrency. The asymptotic distribution of these panel LM statistics follows a standard normal distribution.

While the panel LM unit root test shows structural breaks, the source of this non-stationarity remains clear. We then used the PANICCA test (Reese and Westerlund 2016) to investigate whether the nonstationary source of transaction behaviors and electricity consumption come from common, specific, or common and specific shocks. The PANICCA test combines principal components-based panel analysis of non-stationarity in idiosyncratic and common components and cross-section average test. PANICCA can quantify the driving forces of a series’ non-stationarity and decompose the non-stationarity into shocks of common factors and specific factors. In the model, the data-generating process for the variable is given as follows:

2 $$y_{it} = \phi^{^{\prime}}_{i} D_{t,p} + \eta^{^{\prime}}_{i} G_{t} + u_{it} ,$$

where *y*_*it*_ denotes the concerned variable, including cryptocurrency price and network hashrate; *D*_*t,p*_ denotes a polynomial trend function; *G*_*t*_ represents an r*1 vector of common factors; *η*_*i*_ refers to the corresponding factor loading; and *u*_*it*_ is the idiosyncratic error term. We chose option p = 1 for *D*_*t,p*_, wherein both intercept and trend are included. To test whether cryptocurrency-specific shocks lead to non-stationarity, we used Pap, Pbp, and PMSBp (panel-modified Sargan–Bhargava) tests (Bai and Ng 2004). To test whether a common shock is part of the non-stationarity, we employed the augmented Dickey-Fuller (ADF) test.

If the ADF test rejects the null hypothesis of unit root, the common shock is attributed to non-stationarity. If Pap, Pbp, and PMSBp tests reject the null hypothesis, a specific shock leads to non-stationarity. If all tests reject the null hypothesis, common shocks and specific shocks contribute to non-stationarity. Once structural breaks are examined for both series to test, the long-run cointegration relationship is determined. Notably, the panel cointegration test with structural breaks is utilized as the unit root test considers structural breaks.

### Comovement analysis

To examine the long-run cointegrated relationship between variables (Yang et al. 2022a), we employed the panel cointegration test (Banerjee and Carrion‐i‐Silvestre 2015). Panel cointegration not only explores structural breaks in both the deterministic components and the cointegrating vector but also considers cross-section dependence among units (Yang et al. 2022b; Dey et al. 2022). The data-generating process with structural break form is given as follows:

3 $$Price_{it} = D_{it} + Hash^{\prime}_{it} \delta_{it} + F_{t}^{^{\prime}} \pi_{i} + e_{it} ,$$

where t = 1, … T represents the time period; i = 1, …, N represents units; and *Price*_*it*_ and *Hash*_*it*_ refer to price and hashrate, respectively. Moreover, *F*_*t*_ refers to the common factors; and *e*_*it*_ is the error term. $\delta$_*it*_ denotes break fraction vector. π denotes the loadings of common factor. Panel cointegration test statistics are constructed based on the sum of the individual ADF cointegration test as follows:

4 $$SADF_{\tau } (\lambda ) = \sum\limits_{i = 1}^{N} {t_{{\tilde{e}_{i}^{*} }}^{\tau } }$$

Panel statistics’ limiting distribution follows a standard normal distribution.

### Vector error correction model

Once cointegration can be established, we employed the panel vector error correction model (VECM) to investigate short- and long-run dynamic corrections between cryptocurrency transaction behaviors and electricity consumption (Zheng et al. 2022). We applied a two-step procedure (Engle and Granger 1987). First, we ran the regression in Eqs. (5) and (6) to obtain the residual *u*_*it*_ and *ε*_*it*_ (error correction term; *EM* henceforth).

5 $$Hash_{it} = \alpha_{1i} + \beta_{1i} t + \chi_{1i} Price_{it} + \delta_{1} BP + u_{it}$$

6 $$Price_{it} = \alpha_{2i} + \beta_{2i} t + \chi_{2i} Hash_{it} + \delta_{2} BP + \varepsilon_{it}$$

Here, *BP* refers to the estimated breakpoints in the cointegration test. Second, by incorporating the error correction term, we estimated the following model based on panel Granger causality:

7 $$\Delta Hash_{it} = \theta_{1i} + \lambda_{1i} EM_{it - 1} + \sum\nolimits_{k} {\gamma_{1k} \Delta Hash_{it - k} } + \sum\nolimits_{k} {\varphi_{1k} \Delta Price_{it - k} } + \eta_{1} BP + \omega_{it}$$

8 $$\Delta Price_{it} = \theta_{2i} + \lambda_{2i} EM_{it - 1} + \sum\nolimits_{k} {\gamma_{2k} \Delta Hash_{it - k} } + \sum\nolimits_{k} {\varphi_{2k} \Delta Price_{it - k} } + \eta_{2} BP + v_{it} .$$

By testing the significance of the coefficients of explanatory variables in Eqs. (7) and (8), we can identify short- and long-run causalities between cryptocurrency price and network hashrate. For short-run causality, we check H0:*φ*_*1k*_ = 0 for all k in Eq. (7) or H0:*γ*_*1k*_ = 0 for all k in Eq. (8). Hence, we test the significance of the speed of adjustment λ to examine long-run causality (Omonijo and Zhang 2022; Maiti 2022). For long-run causality, we test H0: *λ*_*1i*_ = 0 for all *i* in Eq. (7) or H0: *λ*_*2i*_ = 0 for all *i* in Eq. (8). Moreover, we utilize the joint test to analyze long-run causality (Jiang et al. 2022; Feng and Zheng 2022).

**Fig. 1:**
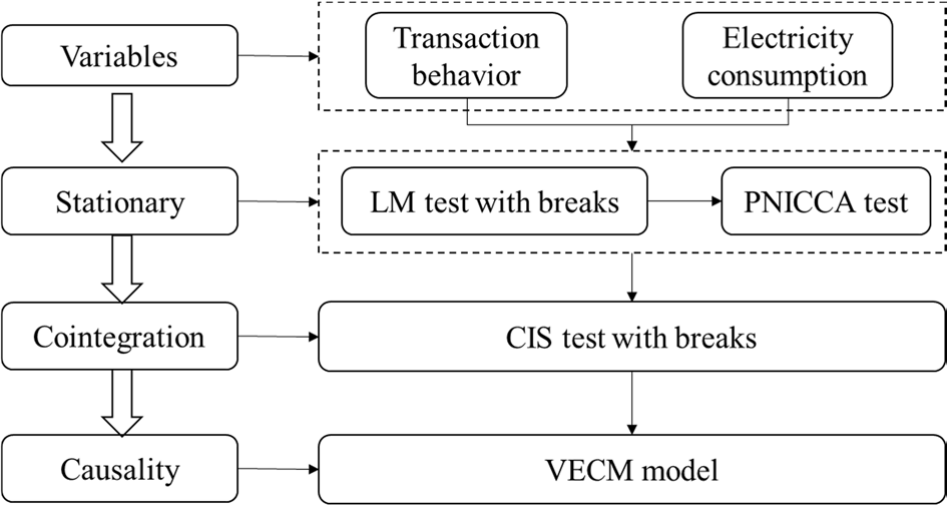
the empirical process in this paper

Figure 1 illustrates the empirical test process. First, we used the panel unit root test with breaks to check the stationarity of the series of transaction behaviors and electricity consumption. After identifying potential breaks, we use the PANICCA test to find the source of external shocks. We then apply the panel cointegration test with breaks to explore the long-run equilibrium relationship between cryptocurrency transaction behavior and electricity consumption. Finally, a cointegrated relationship exists, we then use the VECM model to discover short- and long-run causalities between these two variables (Lee and Hussain 2022; Hao et al. 2022).

### Data sources

We use daily data of 13 cryptocurrencies from July 31, 2015 to July 12, 2019 to analyze the long-run integrated relationship between cryptocurrency transaction behaviors and electricity consumption (Table 1). Particularly, we use market price to proxy transaction behavior and network hashrate to proxy electricity consumption. This is because higher price is positively associated with more transactions, and the electricity consumption of mining is calculated based on network hashrate. Owing to positive cointegration between hashrate and electricity consumption, Schinckus et al. (2022) argue that the former well reflects cryptocurrency electricity consumption. As our methodology only applies to balanced panel data, these coins were selected based on data availability and completeness.

**Table 1:** Selection of cryptocurrency

Cryptocurrency	Price(US$)	Hashrate(KH/s)	Market capitalization (US$)
Bitcoin	11,583	6.732e+19	2.063e+11
Blackcoin	0.0526	6.943e+13	4,083,737
Dash	144.363	3.722e+15	1.288e+09
Dogecoin	0.003	4.249e+14	3.995e+08
Ethereum	273.617	1.793e+14	2.924e+10
Feathercoin	0.020	5.914e+09	4,146,440
Litecoin	104.539	4.742e+14	6.550e+09
Monero	91.675	3.069e+08	1.567e+09
Namecoin	0.866	5.649e+19	14,017,153
Novacoin	0.583	1.859e+11	1,498,238
Peercoin	0.352	1.173e+16	8,968,640
Reddcoin	0.002	6.433e+09	47,995,389
Vertcoin	0.399	6.875e+11	20,096,402

The price, network hashrate, and market capitalization of 13 cryptocurrencies on July 12, 2019. Price and hashrate take the average over the full time span

As our methodology required constructing a balanced panel, the analyzed period ranged from July 31, 2015 to July 12, 2019. We obtained cryptocurrency price data and network hashrates from https://bitinfocharts.com. Cryptocurrency’s price is measured by average price (exchange rate) per day in US dollars. Our sample enjoys good representativeness as the capitalization of the selected cryptocurrencies considers 80% of total global cryptocurrency capitalization on July 12 2019. The analysis utilized the natural logarithm of cryptocurrency price and network hashrate.

Table 2 offers descriptive statistics about *Price* and *Hashrate*. We found that among the 13 cryptocurrencies, the mean values of *Price* and *Hashrate* for Bitcoin are highest. Similarly, Bitcoin has the largest standard deviation, suggesting that *Price* and *Hashrate* are widely distributed in the series of Bitcoin. The mean *Price* values for Dogecoin and Reddcoin are the lowest at 0.002, implying that these two cryptocurrencies are underdeveloped compared to others.

**Table 2:** Summary statistics

Crypto	Variable	N	Mean	SD	Min	Max
Bitcoin	*Price*	1443	3881.961	3813.727	213.673	19,401.000
	*Hashrate*	1443	1.776e+19	1.976e+19	3.183e+17	7.261e+19
Blackcoin	*Price*	1443	0.125	0.153	0.021	1.108
	*Hashrate*	1443	7.170e+13	1.787e+13	3.754e+13	1.172e+14
Dash	*Price*	1443	164.554	226.763	2.088	1436.000
	*Hashrate*	1443	9.259e+14	1.132e+15	5.579e+10	4.011e+15
Dogecoin	*Price*	1443	0.002	0.002	0.000	0.016
	*Hashrate*	1443	8.488e+13	1.097e+14	9.600e+11	4.422e+14
Ethereum	*Price*	1443	205.027	255.812	0.444	1356.000
	*Hashrate*	1443	1.012e+14	1.033e+14	5.138e+10	2.959e+14
Feathercoin	*Price*	1443	0.060	0.095	0.003	0.612
	*Hashrate*	1443	2.619e+09	2.591e+09	31,340,743.000	1.335e+10
Litecoin	*Price*	1443	50.439	61.222	2.696	352.799
	*Hashrate*	1443	1.019e+14	1.287e+14	9.706e+11	4.742e+14
Monero	*Price*	1443	71.068	88.909	0.369	439.391
	*Hashrate*	1443	2.454e+08	2.640e+08	9,946,912.000	1.075e+09
Namecoin	*Price*	1443	1.004	0.949	0.174	7.442
	*Hashrate*	1443	1.317e+19	1.534e+19	8.562e+16	6.487e+19
Novacoin	*Price*	1443	2.030	2.048	0.378	11.354
	*Hashrate*	1443	1.568e+11	1.610e+11	1.072e+10	9.714e+11
Peercoin	*Price*	1443	1.098	1.166	0.211	9.118
	*Hashrate*	1443	1.511e+16	1.195e+16	7.960e+14	5.881e+16
Reddcoin	*Price*	1443	0.002	0.003	0.000	0.029
	*Hashrate*	1443	5.996e+09	2.785e+09	1.628e+09	1.686e+10
Vertcoin	*Price*	1443	0.894	1.607	0.019	9.386
	*Hashrate*	1443	7.207e+11	1.069e+12	4.026e+09	5.427e+12
Total	*Price*	18,759	336.790	1476.382	0.000	19,401.000
	*Hashrate*	18,759	2.380e+18	8.947e+18	9,946,912.000	7.261e+19

## Results

### Stationary and external shocks in transaction behaviors and electricity consumption

**Fig. 2:**
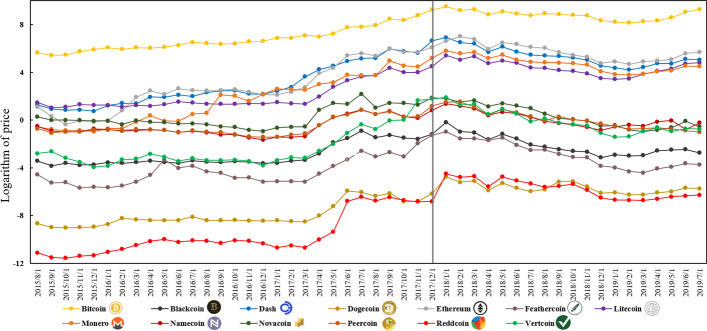
Price evaluation of 13 cryptocurrencies. *Notes*: We choose the price of each cryptocurrency on the first day of each month to represent the monthly price. Daily data are used in the remaining parts of this paper

Our analysis employed the logarithm of cryptocurrency price (*Price*, in USD) and of the network hashrate (*Hashrate*). As a comovement relationship cannot established in an unstable series, we first examined the stationarity of the *Price* and *Hashrate* series over three subperiods: the full subperiod of 7/31/2015–7/12/2019 and the two subperiods representing before and after the peak price date, December 17, 2017 (Ciaian et al. 2018; Corbet et al. 2018). We considered these two subperiods as most cryptocurrencies reversed following that day. Before the peak price date, prices stayed on an upward trend but then significantly pulled back (Fig. 2) after the date, leading to the *Price*–*Hashrate* nexus varying in different time periods.

When examining the stationarity of *Price* and *Hashrate*, addressing structural breaks improperly will lead to biased inferences owing to bull and bear markets in cryptocurrencies (Cheah and Fry 2015). One structural break involved the price bubble of April 2017 accompanied by the passing of a recognition law for Bitcoin as official payments in Japan. Moreover, structural breaks are also essential in modeling series in mature financial markets (Bouri et al. 2019; Mensi et al. 2019; Thies and Molnár 2018; Palamalai et al. 2021). Hence, we addressed these breaks in the model.

We noted that both *Price* and *Hashrate* series follow a stationary process for the whole period 7/31/2015–7/12/2019 and its subperiods (divided by 12/17/2017) after considering potential external shocks. This reflects the existence of external shocks, and the common price bubbles in December 2017 do not affect the series’ stationarity. We found that both *Price* and *Hashrate* for each cryptocurrency were stationary in each subperiod, suggesting that daily shocks do not affect the mean-reverting behaviors of *Price* and *Hashrate*. This implies that price and network hashrate show stable growth trends in the cryptocurrency market after considering breaks. Our results corroborated Bariviera (2017), Sensoy (2019), López‑Martín et al. (2021) and Kang et al. (2022) who support the argument that the cryptocurrency market has become more efficient over time. Charfeddine and Maouchi (2019), Lahmiri et al. (2018), Aggarwal (2019), and Aharon and Qadan (2019) also found persistence in several cryptocurrency market through ordinary least squares and generalized autoregressive conditional heteroskedasticity models but may have neglected the disturbance of structural breaks. Bai and Perron (2003) note that structural breaks can result in misleading results relative to stationarity. Owing to peer-to-peer transactions and anonymous trading (Fang et al. 2022), fluctuations in the cryptocurrency market are drastic. Applying digital technologies and machine learning approaches in cryptocurrency trading also leads to frequent price jumps for cryptocurrencies (Wang et al. 2022b; Sebastião and Godinho 2021). Hence, structural breaks exist in the cryptocurrency market. Additionally, our results showed the importance of structural breaks in testing the stationarity of cryptocurrency.

Table 3 presents the effects of structural breaks on the inherent trend (intercepts) for *Price* and *Hashrate* over the full subperiod. For *Price*, while 3/13 cryptocurrencies exhibited insignificant breakpoint effects, most cryptocurrencies experienced structural change induced by external shocks (e.g., government claims on cryptocurrency or production mechanism changes). Ethereum witnessed a positive external shock in its price series on February 11, 2016 owing to the upcoming birth of the new version “Homestead” in the second hard fork of Ethereum. Moreover, Dash enjoyed a sharp price growth on April 9, 2017 owing to cooperation between Dash and digital payments service BlockPay and speculative activity. Additionally, Bitcoin’s price experienced a negative shock on April 9, 2017, which is accompanied by the People’s Bank of China’s declaration that Bitcoin is not a circulated currency but a commodity (Su et al. 2018). Of the 10 cryptocurrencies with significant shocks, 40% and 60% witnessed negative and positive shocks, respectively. Therefore, most cryptocurrencies remain on the way toward transaction growth.

**Table 3:** Results of the panel LM unit root test with one break

Samples	Price						Hashrate					
	Full sample		Before 2017/12/17		After 2017/12/17		Full sample		Before 2017/12/17		After 2017/12/17	
	Break date	DB	Break date	DB	Break date	DB	Break date	DB	Break date	DB	Break date	DB
Bitcoin	2017/4/9	− 0.066**	2017/3/28	− 0.052*	2019/5/14	− 0.039**	2017/8/22	− 0.033	2016/1/18	− 0.088	2018/10/22	− 0.259***
Blackcoin	2017/9/14	− 0.084**	2017/3/30	0.091**	2018/11/24	0.021	2017/6/9	− 0.073	2017/1/26	0.066	2019/2/2	0.012
Dash	2017/4/3	0.179***	2017/2/14	0.072*	2019/3/31	− 0.038*	2017/9/29	0.043	2017/9/19	0.106	2018/7/4	− 0.070
Dogecoin	2017/11/23	− 0.143***	2017/9/12	− 0.001	2018/8/30	0.218***	2016/11/5	− 0.346***	2016/8/31	0.092	2018/10/21	− 0.085*
Ethereum	2016/2/11	0.152***	2016/1/25	0.021	2018/9/1	0.003	2016/6/5	− 0.005	2016/3/4	− 0.105**	2018/10/21	− 0.018
Feathercoin	2017/11/19	0.339***	2016/7/20	− 0.363***	2019/3/7	− 0.033	2018/11/30	− 0.037	2016/4/21	0.037	2018/8/30	− 0.597***
Litecoin	2017/1/30	0.026	2016/1/20	0.023	2019/2/4	− 0.022	2016/10/9	0.080	2017/2/26	0.115*	2018/10/26	0.064
Monero	2016/10/1	0.062	2016/9/17	0.037	2018/12/4	− 0.018	2016/8/31	− 0.033	2016/8/31	− 0.026	2018/4/10	− 0.092
Namecoin	2018/2/24	0.024	2017/3/12	0.449***	2019/5/8	− 0.124***	2017/4/18	− 0.026	2016/7/29	− 0.137*	2018/10/15	− 0.056***
Novacoin	2017/12/15	− 0.122***	2017/3/1	0.196***	2019/5/4	− 0.003	2016/6/3	− 0.143	2016/7/5	0.168	2018/4/16	0.337
Peercoin	2018/8/1	0.077**	2017/9/5	0.109***	2018/11/11	0.077***	2017/9/9	− 0.315**	2017/7/13	0.134	2019/5/1	0.070
Reddcoin	2017/12/20	0.708***	2017/5/19	− 0.253***	2018/5/3	− 0.033	2018/5/29	0.465***	2017/1/25	0.085	2018/2/27	− 0.018
Vertcoin	2017/9/20	0.354***	2017/4/7	− 0.024	2019/2/6	− 0.155***	2017/6/3	0.150	2017/4/8	− 0.125	2018/10/26	0.145
Panel LM	− 11.292***		− 9.715***		− 13.448***		− 9.333***		− 7.890***		− 9.738***	

*, ** and *** show significance at the 10%, 5% and 1% levels, respectively. DB denotes the coefficients in the intercept

Additionally, only 23% of the cryptocurrencies explored (Dogecoin, Peercoin, and Reddcoin) experienced significant external shocks in the *Hashrate* series, including 2 negative shocks and 1 positive shock. Hence, *Price* series are more affected by external shocks versus *Hashrate* series. This may be because large speculative demand induced structural breaks for *Price*, while stable supply mechanism of coin production created less *Hashrate* fluctuation. Another interesting phenomenon is that most external shocks are in 2017, which witnessed price co-explosivity in the cryptocurrency market (Bouri et al. 2019).

After confirming the existence of stationary and external shocks, we next checked sources of common shocks and specific shocks using the recent developed PANICCA test (Reese and Westerlund 2016). PANICCA can help explore the underlying sources of non-stationarity and has been applied to various studies (e.g., efficient market hypothesis) (Hu et al. 2019), energy economics (Feng et al. 2021), and financial economics (Salisu 2019). Table 4 displays sources of external shocks wherein the external shock for the *Price* series changes at different times. The external effect mainly originates from cryptocurrency-specific shocks before December 17, 2017 and from both common and specific shocks after December 17, 2017. Hence, common shocks are becoming more prominent in external shocks for the *Price* series (Beneki et al. 2019; Bouri et al. 2019). For *Hashrate*, external shocks always originate from specific shocks. Hence, cryptocurrency mining behavior is less affected by the market’s common shocks and participants in the market have helped maintain the growth trend.

**Table 4:** Sources of external shock: PANICCA test

Samples	Variable	Common factors	Idiosyncratic component		
		ADF	Pap	Pbp	PMSBp
Before 2017/12/17	*Price*	− 16.985***	0.110	0.126	0.838
	*Hashrate*	− 23.010***	− 3.020	− 1.820	− 1.510
Full sample	*Price*	12.934	− 5.173***	− 3.28***	− 2.076**
	*Hashrate*	− 28.528***	− 0.232	− 0.224	− 0.211
After 2017/12/17	*Price*	4.108	0.620	0.688	0.763
	*Hashrate*	− 23.154***	− 5.861	− 3.465	− 2.005

*, ** and *** show significance at the 10%, 5% and 1% levels, respectively

### Comovement relationship between transaction behaviors and electricity consumption

Once the stationarity of a series is confirmed, the comovement relationship should be explored. A cryptocurrency with a high price tends to have a larger network hashrate and market capitalization on July 12, 2019, implying that *Price* and *Hashrate* are likely to positively correlate (Table 1). For example, Bitcoin was priced at $11,583 and had the largest day-average network hashrate of 67.36 exahashes per second. Conversely, Reddcoin had a lower price at $0.002 and a network hashrate of 6.43 gigahashes per second. Overall, in our examined period, *Price* seems to positively correlate with *Hashrate*. Hence, *Price* fluctuations are accompanied by *Hashrate* changes during 2015–2019, and *Price* has a comovement trend with *Hashrate*.

Given the existence of external shocks, the above controversial findings may be biased. Thus, we consider external shocks in our comovement analysis. Table 5 shows that *Price* tends to co-move with *Hashrate* in the full sample, and two subsamples once potential external shocks are considered. In the full sample, most break dates (10 out of 13) are found in March 2017–December 2017. This period corresponds to the passing of the law recognizing Bitcoin as an official payment method in April 2017 for Japan, increased attention to cryptocurrencies, and Bitcoin’s hard fork in July 2017 (Bouri et al. 2019). Moreover, we also see that a comovement relationship between *Price* and *Hashrate* is affected by common shocks for all cryptocurrencies. Notably, the break date for Bitcoin is December 16, 2017, which is close to December 17, 2017 when the price of Bitcoin hits its historical peak. This reflects that the comovement relationship between *Price* and *Hashrate* for Bitcoin is affected by external shocks.

**Table 5:** Banerjee and CIS (2015) Panel cointegration test

Cryptocurrency	*Price*-*Hashrate*		
Samples	Full sample	Before 2017/12/17	After 2017/12/17
Panel test	− 5.898**	3.898***	4.754***
Bitcoin	2017/12/6	2017/5/3	2018/11/24
Blackcoin	2017/6/8	2017/6/8	2018/12/11
Dash	2017/3/1	2017/3/1	2018/8/27
Dogecoin	2017/5/20	2016/1/26	2018/8/31
Ethereum	2016/6/17	2016/6/17	2018/9/5
Feathercoin	2016/4/17	2016/4/16	2018/6/23
Litecoin	2017/12/11	2017/3/29	2019/4/2
Monero	2016/8/27	2016/8/27	2018/11/24
Namecoin	2017/3/12	2017/3/12	2018/12/6
Novacoin	2017/9/15	2017/3/15	2018/8/24
Peercoin	2017/11/28	2017/5/3	2018/11/14
Reddcoin	2017/5/21	2017/5/21	2018/8/31
Vertcoin	2017/4/9	2016/1/26	2019/2/16

Panel bayesian information criterion is used to estimate the common factor

***Shows significance at the 1% levels

For the period before December 17, 2017, break dates concentrate over March 2017 to May 2017, which coincides with explosive prices in some cryptocurrencies. For instance, Litecoin and Dash experienced significant price surges in April 2017. For the period after December 17, 2017, break dates are distributed dispersedly, corresponding to weaker interdependence among cryptocurrencies. Similar findings suggest that the break date is close to the date wherein prices change sharply. One example involves Bitcoin’s price drop on November 11, 2018 and the break date on November 24, 2018. This indicates that when predicting *Price* or *Hashrate*, market investors should note possible market changes even if a valid comovement relationship exists between transaction behavior and electricity consumption.

Our results on the comovement between cryptocurrency transaction behaviors and electricity consumption are partly in line with Hayes (2017), Fantazzini and Kolodin (2020) and Mueller (2020). Hayes (2017) showed that hashrate has a positive effect on price. Mueller (2020) found that Bitcoin miners only responded to negative disequilibria, while Ethereum miners react to disequilibria symmetrically. Fantazzini and Kolodin (2020) state that the Bitcoin price cointegrated with the hash rate during 11/12/2017–24/02/2020 but did not connect with hashrate before 4/12/2017. However, these studies do not consider breaks. With the inclusion of breaks, we confirm a cointegrated relationship between cryptocurrency transaction behaviors and electricity consumption, complementing current literature.

### Short-term and long-term causal effects

*Price* tends to co-move with *Hashrate* during the study’s time period, which means that there is an equilibrium relationship between the two over a long time. That is, once external regime shifts bring a shock or bias to the *Price*–*Hashrate* nexus, the equilibrium will correct this bias and induce it to converge to zero over time. Simultaneously, daily short-run price fluctuations change the profits of mining cryptocurrencies and network hashrates, and vice versa. Hence, as *Price* and *Hashrate* are interdependent, they tend to present a significant dynamic relationship, whether for a day or for a long time.

Table 6 shows the interaction between *Price* and *Hashrate* in the short run with daily shocks and long-run persistent influences. In the short run, *Price* significantly affects *Hashrate* in the full sample and the two subsamples. Hence, *Price* fluctuations lead to daily shocks on *Hashrate*. However, in all samples, the causal effect of *Hashrate* on *Price* is insignificant in the short run. Thus, *Hashrate* changes do not critically determine daily *Price* fluctuations. These results are consistent with Fantazzini and Kolodin (2020) and Rehman and Kang (2021) who find unidirectional causality from Bitcoin price to hashrate.

**Table 6:** The causal effect between price and hashrate

Dependent variable	Short run		Long run		
	Δ*Price*	Δ*Hashrate*	λ	λ/Δ*Price*	λ/Δ*Hashrate*
Full sample					
Δ*Price*		1.86	− 0.001		1.25
Δ*Hashrate*	104.95***		0.001**	72.31***	
Before 2017/12/17					
Δ*Price*		0.95	0.00001		0.63
Δ*Hashrate*	91.32***		0.0005**	63.22***	
After 2017/12/17					
Δ*Price*		2.21	− 0.00004		1.50
Δ*Hashrate*	14.03***		0.00003	9.37***	

*, ** and *** show significance at the 10%, 5% and 1% levels, respectively

When turning to the long run with the permanent case, *Price* has a significant causal effect on *Hashrate* in the full sample and subperiod before December 17, 2017. This reflects that price shocks result in a permanent effect on network hashrates. Nevertheless, causal effects of *Price* on *Hashrate* disappear in the subperiod after December 17, 2017. Thus, *Hashrate* is gradually less affected by *Price*. Additionally, the causal effects of *Hashrate* on *Price* do not appear long term. This implies that shocks from *Hashrate* on *Price* gradually vanish and prices eventually return to their permanent equilibrium level.

## Discussion and conclusion

Unprecedented transactions on growth and market capitalization in the cryptocurrency market along with the possibility of considerable profit have increased the interests of financial investors, mainstream media, speculators, and regulators on cryptocurrencies. Hence, understanding the underlying driving forces of cryptocurrency transaction dynamics has attracted greater attention from academia.^2^ Possible macroeconomic, technological, or other influential factors including internet search queries, gold prices, financial stress, hashrate, and trade volume have been explored (Kristoufek 2013, 2015). We contribute to this strand of literature by investigating the interactive relationship between electricity consumption and cryptocurrency transactions dynamics. We prove that electricity consumption to transaction behaviors do not have a causal direction, regardless of daily or longer time periods and network hashrates failing to drive cryptocurrency prices. However, transaction behavior comoves with electricity consumption under possible external shocks in the long term, helping investors or regulators predict future transactions trends by noting electricity consumption under the sign of common market shocks.

Moreover, we show that transaction behaviors have causal impact on electricity consumption for both daily and longer time periods. Hence, electricity consumption do not solely respond to daily transactions changes, but their impacts evolve into persistent shocks. As electricity consumption is closely associated with energy consumption and greenhouse gas emissions (Chai et al. 2022; Ren et al. 2022; Xue et al. 2022; ), environmental organizations and governments should prioritize the promoting effect of cryptocurrency prices on these two (Luo et al. 2022; Hao et al. 2023). Notably, many studies in the literature argue that cryptocurrency mining consumes much electricity and generates considerable CO_2_ emissions (Foteinis 2018; Krause and Tolaymat 2018; Mora et al. 2018; Stoll et al. 2019). As transaction behaviors exert persistent effects on electricity consumption and lead to environmental costs, regulators should revisit their attitudes toward cryptocurrency development.

Our results contain implications for developing market investment strategies. Overall, cryptocurrency transactions return to their equilibrium trend in the long term after facing temporary shocks, and the price series show mean-reverting behavior. While external shocks may exist in price series, structural breaks are short-lived and do not affect the stationary characteristics. Stationarity implies that market participants (e.g., cryptocurrency miners or speculators) have a limited role in determining price trends. Thus, market investors need not adjust their investment excessively after daily shocks or weekly shocks. Moreover, market investors could use such hidden information to construct a strategy to obtain more profits.

Future research can employ other advanced methodologies to identify the cointegration nexus of cryptocurrency electricity consumption and price (Ferreira et al. 2020). A detrended cross-correlation analysis can identify integration for nonstationary series (Ferreira et al. 2016). Detrending moving-average cross-correlation analysis can also present the correlation for possible nonstationary series (Kristoufek 2014). As Demir et al. (2020) found a cointegration between cryptocurrencies and COVID‑19 (Wang et al. 2021; Long et al. 2022), investigating the effect of COVID-19 on cointegration between cryptocurrency electricity consumption and price would be of interest.

## Data Availability

The datasets used and/or analysed during the current study are available from the corresponding author on reasonable request.

## Abbreviations

ADF: Augmented Dickey-Fuller
BP: Break point
CA: Cross-section average
COVID: Corona Virus Disease
DCCA: Detrended cross-correlation analysis
DMCA: Detrending moving-average cross-correlation analysis
GARCH: Generalized AutoRegressive Conditional Heteroskedasticity
LM: Lagrange Multiplier
OLS: Ordinary least squares
PANIC: Principal components-based panel analysis of non-stationarity in idiosyncratic and common components
PANICCA: Principal components-based panel analysis of non-stationarity in idiosyncratic and common components, and cross-section average
PMSB: Panek modified Sargan–Bhargava
TB: Time break
VECM: Vector error correction model

## Footnotes

1 Digiconomist is a platform that provides reports, data and articles of digital trends. In particular, Digiconomist provides energy consumption and carbon footprint data on multiple crypto assets, including Bitcoin, Ethereum, and Dogecoin.

2 Xu et al. (2019) provide a systematic review on research progress in blockchain and cryptocurrency.
